# Targeting regulatory T cells in cytokine-induced killer cell cultures (Review)

**DOI:** 10.3892/br.2014.234

**Published:** 2014-02-07

**Authors:** QIANSHAN TAO, HUIPING WANG, ZHIMIN ZHAI

**Affiliations:** Department of Hematology, The Second Affiliated Hospital of Anhui Medical University, Hefei, Anhui 230601, P.R. China

**Keywords:** regulatory T cells, cytokine-induced killer cells, adoptive immunotherapy

## Abstract

Regulatory T cells (Tregs) are potent immunosuppressive cells that promote tumor growth and invasion by inducing immune escape and suppressing the antitumor immune response. Cytokine-induced killer (CIK) cells are considered to be the primary candidate for adoptive immunotherapy due to their strong antitumor activity. It was recently reported that the concomitant presence of Tregs may decrease the cytotoxicity of CIK cells. Therefore, depletion or downregulation of Tregs in CIK cell cultures by optimizing the culture program may enhance CIK cell cytotoxicity *in vitro* and *in vivo*. The aim of the present review was to summarize the currently available studies on the optimal culture strategy for improving the antitumor activity of CIK cells through targeting Tregs.

## 1. Introduction

Regulatory T cells (Tregs) are potent immunosuppressive cells that are essential for inducing immune tolerance and preventing autoimmune and inflammatory diseases (1,2). However, Tregs are commonly increased in malignancies and may limit beneficial responses by suppressing sterilizing and antitumor immunity (3,4). For this reason, finding effective methods to neutralize Tregs or their function is critical for successful tumor immunotherapy. Moreover, cytokine-induced killer (CIK) cells comprise heterogeneous cell populations, including a major effector cell population expressing both T-cell and natural killer (NK)-cell markers and lyse target cells in a non-major histocompatibility (MHC)-restricted manner (5). CIK cells were shown to be a promising tool in antitumor adoptive immunotherapy strategies (6); however, the main functional properties of CIK cells may be limited by certain inhibitory factors. It was recently reported that the concomitant presence of Tregs in CIK cell cultures may decrease CIK cell cytotoxicity, whereas depletion or downregulation of Tregs by optimizing the culture program may improve the antitumor activity of CIK cells *in vitro* and *in vivo*. The present review aimed to summarize the currently available studies on the optimal culture strategy for improving the antitumor activity of CIK cells through targeting Tregs.

## 2. CIK cell immunotherapy and Tregs

Adoptive immunotherapy, a potential new approach, holds great promise in the treatment of various tumors that may be refractory to conventional therapies. Schmidt-Wolf *et al* (5) were the first to report that CIK cells, which are now considered as the primary candidate for adoptive immunotherapy, exert strong antiproliferative and cytotoxic effects against tumor cells (5,6). CIK cells are cytotoxic immune effector cells that are readily expandable and express the T-cell marker CD3, as well as the NK-cell marker CD56. The cytotoxicity of CIK cells is MHC-unrestricted and T-cell receptor-independent (5,7,8). The exact mechanisms underlying tumor cell recognition and elimination have not been fully elucidated in CIK cells; however, the NK cell-activating receptor NK group 2 member D (NKG2D), which is expressed on the membrane of CIK cells and interacts with MHC-unrestricted ligands on tumor cells, may play a predominant role (9). CIK cells may be generated by *in vivo* culture of peripheral blood mononuclear cells from healthy donors or tumor patients with interferon (IFN)-γ, interleukin (IL)-2, IL-1 and anti-CD3 monoclonal antibodies (mAb) (5). CIK cells may also be generated by incubating mononuclear cells from the bone marrow or cord blood with various types of additions (10,11).

The relatively robust and simple cell culture procedures used to expand CIK cells have enabled antitumor adoptive immunotherapy to be increasingly investigated worldwide. Numerous attributes for the use of CIK cells were developed over the past 2 decades. Clinical studies on CIK cells confirmed the benefits and safety for patients with hematological malignancies and solid tumors (10,12). The International Registry on CIK Cells was recently established to collect data worldwide and set standard criteria to report the results of clinical trials performed with CIK cells (13).

It was also demonstrated that combining chemotherapy, radiotherapy or other immunotherapy approaches with CIK cells may further enhance the therapeutic effect and prolong the survival of cancer patients (6,14). Moreover, enhancing the potency and specificity of CIK cell immunotherapy via optimizing the culture program may significantly improve their antitumor activity. In particular, it was reported that Tregs decreased the cytotoxicity of CIK cells (15), whereas cytotoxicity was enhanced when Tregs were removed or downregulated. The *in vitro* culture of CIK cells exhibited strong induction of CD4^+^CD25^+^ cells with high secretion of IL-10 following unspecific stimulation of the T-cell receptor (TCR) complex and IL-2 (15). Depletion of CD25^+^ cells resulted in increased cytotoxicity and reduced IL-10 release in CIK cells. Furthermore, depletion of CD25^+^ cells pre-culture significantly increased the proliferation and antitumor activity of CIK cells *in vivo* and *in vitro* (15). Transforming growth factor-β (TGF-β) and glucocorticoid-induced tumour necrosis factor receptor-related protein may participate in the immune regulation of Tregs in CIK cell cultures and the inhibition of these two molecules was found to partially abrogate the inhibitory effects of Tregs on CIK cell proliferation and cytotoxicity (15).

## 3. Targeting Tregs in CIK cells

### Dendritic cell (DC)-CIK cells

Following co-culture of CIK cells with DC (DC-CIK cells), Tregs, the expression of TGF-β and IL-10 were downregulated; however, the main effector cells, including CD3^+^CD56^+^ NKT cells, cytokine expression and cytotoxicity were all significantly upregulated (16,17). Moreover, T-bet, as a transcription factor controlling IFN-γ expression in T helper 1 cells, further enhanced the antitumor effects of DC-CIK cells by suppressing Treg pathways (18). Thus, DC-CIK or improved DC-CIK cells may be used for the induction of a specific immune response by blocking the properties of Tregs and Treg-related cytokines.

### Thymoglobulin (TG)-CIK cells

TG is a purified, pasteurized preparation of polyclonal rabbit γ-immunoglobulin directed against human thymocytes and displays specificity towards a wide variety of surface antigens in the immune system. CIK cells are typically generated with anti-CD3 mAb and other cytokines; however, a previous study reported that TG fostered the generation of functional CIK cells with no concomitant expansion of tumor-suppressive Tregs compared to anti-CD3 mAb (19).

### IL-6-CIK cells

Over the last few years, the potential effects of other cytokines on the generation of Tregs during the preparation of CIK cells were investigated. A previous study assessing the proportion of Tregs in CIK cells cultured with and without IL-6 revealed that IL-6 improved the proliferation and cytotoxic activity of CIK cells. However, the proportions of Treg/CD4^+^ and Treg/CD3^+^ cells were decreased in IL-6-CIK cells, suggesting that the addition of IL-6 during CIK cell culture *in vitro* inhibited the production of Tregs (20).

### IL-2-CIK cells

As mentioned above, CIK cells are typically generated by *in vitro* culture of mononuclear cells with IL-2, a potent lymphocyte stimulator (5). However, disruption of the IL-2 pathway was shown to result in lymphoid hyperplasia and autoimmunity rather than immune deficiency in mice, indicating that the major function of IL-2 is to limit rather than improve T-cell responses, whereas IL-2 is required for the generation and function of Tregs by upregulating FoxP3 expression (21). Signaling through the IL-2 receptor, in particular, is critical for T-cell differentiation and survival. Tregs express all 3 components of the high-affinity IL-2 receptors (α, CD25; β, CD122; and δ, CD132); however, effector T cells express 2 incomplete components of the low-affinity IL-2 receptors (β, CD122 and δ, CD132) (22). Thus, Tregs may compete with effector T cells for IL-2 and inhibit the proliferation and function of effector T cells (23). Moreover, clinical studies reported that high doses of IL-2 enhance immune responses and exacerbate autoimmune destruction of islets, whereas low doses of IL-2 offer long-lasting protection in the same model, suggesting that the effects of IL-2 vary widely depending on the dosing regimen (24–26).

Therefore, we hypothesized that applying a high dose of IL-2 in CIK cell cultures may enhance the cytotoxicity of CIK cells by selectively expanding effector T cells and inhibiting Tregs *in vitro*. This hypothesis is currently under investigation. In the currently available data, a conventional dose of IL-2 (500 IE/ml) was found to induce the expression of Tregs in CIK cell cultures in a time-dependent manner, with a peak (accounting for ~50% of the total cultured cells) at days 7–8. After ~1 week, the expression of Tregs in CIK cell cultures was gradually decreased with time and was reduced to <5% at day 28; however, CD3^+^CD56^+^ NKT cells and CD3^+^CD8^+^ T cells were significantly increased in CIK cell cultures at the same timepoint. These data suggested that Tregs compete with effector T cells for IL-2 via their high-affinity IL-2 receptor under conditions of IL-2 shortage; however, effector T cells were ultimately able to expand significantly due to the constant addition of IL-2.

Notably, IL-2-based regimens are able to activate cellular antitumor immunity and are the mainstay of immunotherapy directed against tumors (27). IL-7, -15 and -21, in particular, share the common cytokine receptor γ-chain (γ_c_) as well as certain properties with IL-2 and were shown to stimulate innate immunity and increase CD8^+^ T-cell-mediated antitumor activity. Furthermore, the addition of IL-7 or IL-15 was shown to abrogate the suppressive activity of Tregs *in vitro* (28) and the administration of anti-IL-2 plus exogenous IL-15 to tumor-bearing mice enhanced the adoptive immunotherapy of tumors (29). Therefore, we do not consider IL-2 may to be the optimal T-cell growth factor in the culture of CIK cells and other γ_c_-signaling cytokines, such as IL-7, IL-15 and IL-21, may be alternative choices for the optimal culture of CIK cells.

### IL-7-CIK cells

In CIK cell cultures, the presence of IL-7 was shown to abrogate the ability of Tregs to suppress the proliferation of conventional T cells in response to TCR activators, whereas removal of IL-7 restored the suppressive function of Tregs and pre-blocking of the IL-7 receptor on Tregs restored suppressor function (30). Thus, IL-7 was shown to exert a more potent proliferative effect compared to IL-2 in generating CIK cells. Furthermore, IL-7-stimulated CIK cells were more potently cytotoxic (30) and IL-7 gene-transfected CIK cells exhibited an improved proliferation rate and a significantly higher cytotoxic activity compared to non-transfected CIK cells (31).

### IL-21-CIK cells

IL-21-induced CIK cells may be of clinical value in the enhancement of antitumor immunotherapy via increasing the expression of IL-21 receptor, perforin, granzyme B, Fas ligand, IFN-γ and tumor necrosis factor-α, as well as activating the Janus kinase/signal transducers and activators of transcription signaling pathway (32). However, there is currently no published report of IL-21 directly enhancing the cytotoxicity of CIK cells by decreasing Tregs.

### IL-15-CIK cells

IL-15 has attacted increasing attention for use in CIK cell cultures as an alternative to IL-2, as it binds and signals through a complex composed of IL-2/IL-15 common receptors CD122 and CD132 and stimulates CD8^+^T, NK and NKT cell proliferation, survival and effector functions. It was recently reported that IL-15 stimulation resulted in a significant enhancement of CIK cell-mediated cytotoxicity against tumor cells compared to IL-2-induced CIK cells. Further analysis of IL-15-induced CIK cells demonstrated that the NKG2D receptor is also involved in the recognition of target cells and the main effector cells were CD3^+^CD8^+^CD25^+^CD56^−^ cells, which were more effective compared to conventional CD3^+^CD56^+^ cells in the lysis of tumor cells (33,34). In addition, our data (35) demonstrated that the application of IL-15 instead of IL-2 during the generation of CIK cells *in vitro* downregulated the production of Tregs and IL-35, but resulted in a significant enhancement of CIK cell-mediated cytotoxicity against leukemia cells, suggesting that IL-15 may improve the cytotoxicity of CIK cells by inhibiting the production of Tregs and IL-35 expression. Thus, the present review aimed to provide an update on the potential application of IL-15 in the culture of CIK cells and tumor immunotherapy.

## 4. Conclusions

In conclusion, CIK cells are heterogeneous cell populations following *in vitro* expansion and are considered to be the primary candidates for adoptive immunotherapy due to their potent cytotoxicity. However, the concomitant presence of expanded Tregs in CIK cell cultures significantly decreases their cytotoxicity. Depletion or downregulation of Tregs in CIK cell cultures via optimizing the culture program enhances their antitumor activity *in vitro* and *in vivo* and may provide novel strategies for successful tumor immunotherapy.
